# Multiple deprivation and geographic distance to community physical activity events — achieving equitable access to parkrun in England

**DOI:** 10.1016/j.puhe.2020.09.002

**Published:** 2020-12

**Authors:** P.P. Schneider, R.A. Smith, A.M. Bullas, H. Quirk, T. Bayley, S.J. Haake, A. Brennan, E. Goyder

**Affiliations:** a School of Health and Related Research, University of Sheffield, Sheffield, UK; b Centre for Sports Engineering Research, Sheffield Hallam University, Sheffield, UK; c Advanced Wellbeing Research Centre, Sheffield Hallam University, Sheffield, UK

**Keywords:** Parkrun, Physical activity, Health inequalities, Health promotion, Geospatial analysis

## Abstract

**Objectives:**

To evaluate geographic access to free weekly outdoor physical activity events (‘parkrun’) in England, with a particular focus on deprived communities, and to identify optimal locations for future events to further maximise access.

**Study design:**

This study is a cross-sectional ecological analysis of the socio-economic disparities in geographic access to parkrun events in England in late 2018.

**Methods:**

We combined geolocation data on all English Lower Layer Super Output Areas and parkrun events to calculate geodesic distances to the nearest event for more than 32,000 communities in England. We use this measure of geographic access to summarise the relationship between access and socio-economic deprivation, measured using the index of multiple deprivation. We then used geographic coordinates of public green spaces in England to conduct a simple location-allocation analysis to identify 200 locations for future event locations that would maximise access.

**Results:**

In England, 69% of the population live within 5 km of one of the 465 parkrun events. There is a small negative correlation between distance and deprivation, indicating that access is slightly better in more socio-economically deprived areas. Setting up an additional 200 events in optimal locations would improve access: the average distance to the nearest parkrun event would improve by 1.22 km, from 4.65 km to 3.43 km, and approximately 82% of the English population would live within 5 km of a parkrun event.

**Conclusion:**

Over two-thirds of the English population live within 5 km of a parkrun event, and contrary to our expectation, we find that geographic access is slightly better for those living in more deprived communities. Creating additional events may improve geographic access, but effective strategies will still be needed to increase engagement in new and existing events by those living in socio-economically deprived areas.

## Highlights

• Parkrun in a grass-roots movement that organises free weekly 5 km running and walking events.
• In England, the median distance to the nearest parkrun event was 3.4 km; 69% of the population lived within 5 km of an event.
• Access showed no negative socio-economic gradient. In fact, people in deprived communities had better geographic access.
• Sport England provided funding to set up 200 new events. We identified optimal locations, to further maximise public access.

## Introduction

Insufficient physical activity is one of the leading causes of disease and disability worldwide.^1^ In the UK, around one in six deaths is attributable to low levels of physical activity.^2^ It is also a major contributor to health inequalities, as people from low socio-economic backgrounds are both disproportionately likely to be inactive^3^^,^^4^ and be affected by physical inactivity-related diseases.^5^ Increasing the physical activity levels of the population is therefore high on the public health agenda: it not only has the potential to improve quality of life, reduce mortality rates and alleviate the strain on health and social care services but also reduce the gap in health inequalities.^6^

However, designing effective public health interventions that increase population physical activity is a considerable challenge.^7^^,^^8^ Implementing such interventions in a way that does not increase health inequalities might even be more difficult. Studies have shown that programmes to increase physical activity often fail to reach deprived communities and those most in need, suggesting access to physical activity facilities shows spatial and social inequality.^9^^,^^10^ In this regard, parkrun, an international movement which organises free weekly 5 km running and walking events in public spaces, might provide valuable lessons.

Since its founding in 2004, as a small event in London with 13 participants, parkrun has grown to become one of the world's largest mass sporting events, with up to 360,000 participants in more than 20 countries.^11^^,^^12^ The volunteer-led events are often characterised as accessible and inclusive.^13^^,^^14^ The organisation has been widely praised as being successful in encouraging participation particularly in individuals who were previously inactive.^15^^,^^16^

Notwithstanding these subjective accounts, the expansion of parkrun in England, as elsewhere, has been largely grassroots, driven by *demand* rather than *need*. It might therefore be the case that parkrun events are primarily located in areas that are less deprived, while people living in more deprived communities may not have the same opportunities to participate. In 2019, Sport England announced funding to support the creation of 200 new parkrun events across England within three years, with the specific aim of increasing participation of individuals from lower socio-economic groups.^17^

The aims of this study are two-fold: first, to evaluate whether geographic access to parkrun events in England is equitable across areas with different levels of deprivation; and second, to identify 200 optimal locations for future events to improve geographic access, in particular for deprived communities.

## Methods

### Study design

This study is a cross-sectional ecological analysis of the socio-economic disparities in geographic access to parkrun events in England at the end of 2018. All analyses were conducted on the level of Lower Layer Super Output Areas (LSOAs), which divide England into 32,844 geographic units which, on average, have a population of approximately 1700. We assessed the relationship between access, defined as the distance (as the crow flies) to the nearest parkrun event, and socio-economic deprivation, measured using the Index of Multiple Deprivation (IMD). In addition, we used information on public green spaces in England to conduct a simple location-allocation analysis, to identify 200 locations for future parkrun events that maximise access for the population.

### Data sources

For this study, we combined data on three types of geospatial entities: (1) LSOAs, (2) parkrun events, and (3) public green spaces.

1) For all 32,844 LSOAs, we retrieved geographic locations, defined by the coordinates of its population-weighted centroid; 2017 total population estimates; and the 2015 IMD from the Office for National Statistics.^18^, ^19^, ^20^
2) We included all 465 public parkrun events which were in operation in England by December 12th, 2018 – on this date, Sport England announced their plan to provide funding for setting up 200 additional parkrun events across England.^21^ The locations of the events were obtained from the parkrun UK website.^22^
3) The locations of public green spaces in England were retrieved from an open data set of Ordnance Survey.^23^ Parkrun events are held in various settings and terrains and do not always require a single 5 km loop – some events have courses that involve running a combination of shorter loops. After evaluating existing parkrun event courses, we decided to consider all public parks, gardens and playing fields in England with an area of 0.1 km^2^ or more potentially suitable for hosting events (n = 2842).

### Variables

The two variables of interest were access to parkrun events and deprivation of LSOAs.

Access to parkrun was defined as the geodesic distance (as the crow flies) from LSOA's population-weighted centroid to its nearest event. For each of the 32,844 LSOAs, we computed the geodesic distances between its population-weighted centroid and all 465 parkrun events that were in operation on December 12th, 2018 and then selected the shortest distance.

The socio-economic deprivation of LSOAs was measured using the 2015 IMD. It is a measure of relative deprivation, which has been used in many similar studies. The IMD combines 37 indicators from seven domains (income, employment, education and skills, health and disability, crime, housing and services, and living environment) into a single score. The score ranges from 0 (least deprived) to 100 (most deprived).^24^

Other covariates, which are likely to affect the availability of parkrun events within an area (e.g. population density or demographics), were not taken into account because we did not aim to assess to what extent deprivation independently ‘explained’ access. Rather, we sought to evaluate whether or not people living in deprived areas have better or worse geographic access, under the actual circumstances.

### Analysis

Mean, standard deviation, median, interquartile range, and range were used as descriptive statistics. We then assessed the association between the IMD and the distance to the nearest parkrun event on the LSOA level. Our hypothesis was that more socio-economically deprived areas had worse access, i.e. longer geodesic distances to the nearest parkrun event than less deprived areas. Pearson and Spearman correlation coefficients were computed using the LSOAs’ total population as weights. We also conducted a stratified analysis, for which we grouped LSOAs into IMD quintiles (most, more, median, less, least deprived) and assessed access to parkrun events in each stratum.

#### Identifying optimal locations for new parkrun events

We conducted a location-allocation analysis to solve the following problem. Parkrun UK received funding to start 200 additional parkrun events; there are 2842 public green spaces in England in which new events could be set up – which 200 locations should be selected, to maximise access for the greatest number of people?

More specifically, the objective was to minimise the population-weighted total sum of distances between all LSOAs and their nearest parkrun event. To identify the optimal 200 green spaces, we applied a simple greedy algorithm that consisted of two steps. Firstly, for each green space, we evaluate how setting up a parkrun event would affect the sum of distances, given the locations of all existing events (i.e. for how many LSOAs this green space would be the nearest parkrun event, and by how much it would decrease the respective distances). Second, the green space with the greatest effect is selected and added to the set of existing parkrun events. This procedure is repeated 200 times.

More formally, the first step of the algorithm evaluates the following expression:

$$\underset{c\in C}{\mathrm{argmin}}\overset{32,844}{\underset{i=1}{\sum }}d_{i}\left(E\cap c\right)*p_{i}$$

The function yields the candidate green space *c*, from the set of all 2842 green spaces *C*, which minimises the sum of the population-weighted distances between LSOAs and their nearest parkrun event. The total population of LSOA *i* is denoted *p*_*i*_, and *d*_*i*_(*E* ∩ *c*) denotes LSOA *i*'s distance to the nearest parkrun event, which can either be an existing event from the set of 465 parkrun events, denoted *E*, or the candidate green space *c*, whichever is nearest.

To identify the optimal new locations for setting up 200 new parkrun events consecutively, the selection procedure is repeated 200 times. At each step, the single best candidate green space location is selected, added to the set of established parkrun events *E* and removed from the set of available green spaces *C*. This means, the effect of the green space selected at step *k* is taken into account when selecting the *k*th+1 location.

We assessed the overall impact of setting up 200 new parkrun events on the geographic access to parkrun events in England. We also investigated the effects on LSOAs across IMD quintiles in a distributional analysis.

### Data and source code availability

All data and the R source code that were used to generate the results of this study are provided on an open repository.^25^ Ethical approval

Ethical approval was obtained from the Sheffield Hallam University Ethics Committee (ER10776545). We did not collect any personal information, and only used aggregate secondary data. The parkrun Research Board approved this research project, and four of its members (A.M.B., H.Q., E.G., SS.J.H.) were actively involved in the interpretation of findings and writing of this manuscript.

## Results

### Descriptive statistics

As of 12th December 2018, approximately 7%, 69%, and 91% of the English population lived within 1, 5, and 10 km distance of a parkrun event, respectively. Only 578,043 people (1% of the English population) lived more than 20 km from an event. The mean (standard deviation (SD)) and median (interquartile range (IQR)) distance to the nearest parkrun event were 4.65 (4.22) and 3.39 (1.99–5.83) km. The largest distance was observed for the 2259 people living on the Isles of Scilly, who live about 76 km away from the next parkrun event on the mainland. On average, each parkrun event is the closest event for 71 LSOAs (43), with a combined population of 119,612 (74,290). Further descriptive statistics are provided in Table 1.

**Table 1:** Descriptive statistics of LSOAs and parkrun events.

Variable	Mean (SD)	Median (Q25-Q75)	Range
**LSOAs (n = 32,844)**			
Population	1693 (405)	1612 (1452; 1834)	362-13,404
IMD	21.67 (15.59)	17.40 (9.65; 30.07)	0.48–92.60
Distance (in km) to the nearest event	4.65 (4.17)	3.39 (1.99; 5.83)	0.04–76.44
**Parkrun events (n = 465)**			
Catchment area^a^ population	119,612 (74,290)	103,952 (68,837; 151,488)	7855–628,010
Catchment area^a^ LSOAs	71 (43)	62 (40; 87)	6–350

a Number of LSOAs/total population for which a given parkrun event is the nearest.

### Association between deprivation and access

There was a negative relationship between IMD and the distance to the nearest parkrun event: the (population-weighted) Pearson and Spearman correlation coefficients were −0.15 and −0.18, indicating a small negative correlation. This means that more deprived LSOAs tended to have shorter distances to the nearest parkrun event, i.e. better geographic access.

The analysis of distances by IMD quintile in Table 2 shows that people living in the 20% most deprived LSOAs had the best geographic access, with a mean and median distance to the nearest parkrun event of 3.51 and 2.79 km, respectively. Depending on the metric, the worst access was observed for LSOAs in the middle (mean distance = 3.36 km) or the less deprived group (median distance = 3.93 km). Further results of the distributional analysis are provided in Table 2.

**Table 2:** Distributional analysis. The table shows the distance (in km) to parkrun events before and after 200 new parkrun events are set up at optimal green spaces, stratified by IMD quintiles.

	Current situation (December 12th, 2018)			After 200 new parkrun events are set up		
Variable	Mean (SD)	Median (Q25-Q75)	Range	Mean (SD)	Median (Q25-Q75)	Range
Least deprived	4.93 (3.62)	3.91 (2.27; 6.67)	0.12–58.54	3.79 (2.61)	3.09 (1.92; 4.96)	0.12–25.58
Less deprived	5.21 (4.24)	3.93 (2.28; 6.99)	0.14–76.44	3.92 (3.04)	2.99 (1.84; 5.09)	0.14–48.02
Median deprived	5.36 (5.01)	3.68 (2.12; 6.83)	0.11–60.81	3.98 (3.55)	2.79 (1.70; 4.91)	0.11–33.74
More deprived	4.26 (4.38)	2.96 (1.76; 5.00)	0.04–59.44	3.03 (2.78)	2.27 (1.47; 3.49)	0.04–24.07
Most deprived	3.51 (3.01)	2.79 (1.71; 4.39)	0.07–36.17	2.43 (1.68)	2.12 (1.41; 3.02)	0.05–24.30
Overall	4.65 (4.17)	3.39 (1.99; 5.83)	0.04–76.44	3.43 (2.86)	2.59 (1.63; 4.16)	0.04–48.02

### Optimal locations for new parkrun events

**Fig. 1:**
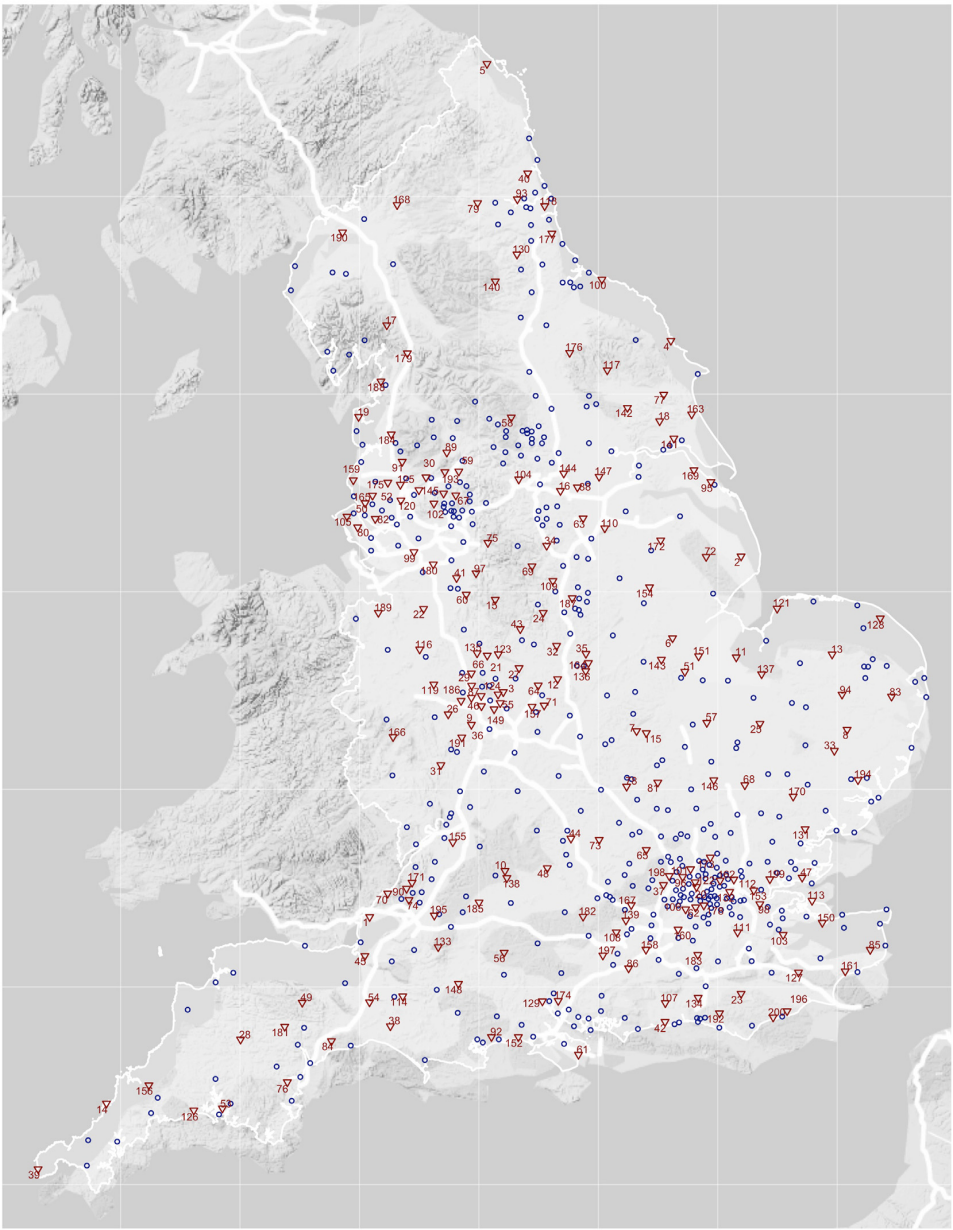
Map of England showing current parkrun events (blue circles) and recommended new event locations (red triangles) ranked in descending order of estimated effect on overall population-weighted access. Information on all 200 identified optimal green space locations are provided in the appendix. (For interpretation of the references to colour in this figure legend, the reader is referred to the Web version of this article.)

Fig. 1 shows the parkrun events (blue circles) that existed on 12th December 2018 alongside recommendations for 200 additional event locations (red triangles), which minimise the sum of the population-weighted geodesic distances from the LSOA centroids, i.e. maximise overall access to parkrun for the greatest number of people. The numbers correspond to the rank, where 1 is the location which would improve access the most. The names and exact locations of the selected 200 green spaces are provided in Table S1 in the appendix. We also created an interactive map, which can be accessed online, to explore the locations of existing and recommended parkrun event locations in more detail: https://bitowaqr.github.io/parkrun_access_equity/.

We estimated that setting up new parkrun events in those 200 green spaces would improve access for around 16.5 million people (30% of the population) from 9854 LSOAs. For these people, the distance to the nearest event would, on average, be reduced by 4.09 km (SD = 3.97). Overall, it would reduce the average and median distance to the nearest parkrun event from 4.65 and 3.39 km to 3.43 and 2.59 km. The percentage of people who live within 5 km of a parkrun would increase from 69% to 82%.

The distributional analysis in Table 2 shows, for each IMD quintile, geographic access under the current situation (12th December 2018) and after the creation of 200 new events. Overall, setting up 200 new events in the recommended green spaces would amplify the negative socio-economic gradient in geographic access. The population-weighted Pearson and Spearman correlation coefficients changed from −0.15 and −0.18 before, to −0.20 and −0.23 afterwards, indicating that improvements in access to parkrun events were greater for more deprived LSOAs than less deprived LSOAs. Nevertheless, the distributional analysis showed that the improvement in access was smallest for LSOAs in the most deprived quintile.

## Discussion

As of 12 December 2018, the median distance to the nearest parkrun event was 3.39 km and more than two-thirds of the English population lived within 5 km (the parkrun distance) of a parkrun event. Contrary to our expectation, we did not find that access was better for people living in less deprived areas. In fact, those living in the most deprived areas had the best geographic access to parkrun – it is rare in public health for inequalities to exist in this direction.^10^^,^^26^

Our analysis has shown that setting up 200 new events in the recommended (optimal) green spaces in England would reduce the average distance to the nearest parkrun event by 1.22 km, increasing the percentage of English residents who live within 5 km of a parkrun to 82%. Moreover, the recommended expansion of parkrun would improve the geographic access for the most deprived areas more than the access of those living in more affluent areas.

The main finding, that geographic access to parkrun events is better in more deprived communities, is surprising. Parkrun events are set up by volunteers, based on demand not need. Studies have shown that the level of physical activity, and the availability of physical activity facilities generally declines with the level of deprivation.^4^ Opportunities for physical activity are often lacking in areas in most need.^27^ Parkrun events, in contrast, seem to be often held in or near deprived areas, and are free to attend, giving anyone equal access, irrespective of their socio-economic background. Nevertheless, in a previous analysis, we found that participation in parkrun has a strong socio-economic gradient with considerably higher participation rates in less deprived areas: about a third of all participants came from LSOAs in the least deprived quintile, whereas only 7% came from the most deprived quintile.^28^^,^^29^ This suggests that providing the opportunity to participate in parkrun events, while a necessary first step to enable participation, has not been sufficient to engage people living in deprived communities.^9^ This means, creating additional events in optimal locations could improve overall geographic access further, yet effective strategies will still be needed to increase equity in engagement in new and current events.

There are several strengths of this study that deserve mention. First, it is the first study of geographic access to parkrun in England – therefore the approach is novel and the data untapped. Second, the analysis makes use of large and rich data sets, with more than 30,000 LSOA and more than 400 existing parkrun events; it is unlikely that individual outliers are affecting the results. The almost universal availability of parkrun events throughout the country provides a learning opportunity to explore socio-spatial determinants influencing physical activity behaviour on a national scale.^16^ Our study contributes to the limited research in this area and identifies possible leads for further investigation.

However, there are also limitations. Most importantly, geographic access is not measured as travel distance, or travel time, but as geodesic distance. In some cases, for example, where natural barriers such as hills, lakes or rivers block routes, the actual distance travelled may be far in excess of the geodesic distance.^30^

Furthermore, the list of green spaces that we considered as potential sites for future parkrun events is neither comprehensive nor without limitations. Not all included green spaces may be suitable to host events (e.g. because of the terrain or the setting), and the list also does not contain all suitable places (e.g. many blue spaces such as beaches and promenades are not included).

Finally, our analysis has been concerned only with determining to what extent deprived communities have geographic access to parkrun events. We did not investigate what other factors independently explain access more generally. It should be noted, however, that a contributing factor for the negative relationship between IMD and access is likely to be population density: deprived areas cluster in urban areas, where also most parkrun events take place. Rural areas, on the other hand, may therefore have worse geographic access. Further studies are required to better understand wider determinants of access to parkrun and/or physical activity facilities more generally.

Studying barriers to participation in parkrun, other than geographic access, is likely to improve our understanding of the reasons why physical activity levels are lower in more deprived areas and may help to design more effective public health interventions to increase levels of physical activity in the population. Future research should build on this work and develop a model to assess the (cost-)effectiveness of setting up new events, and other strategies, not only in terms of improved potential access but also actual participation. This requires estimating the causal and marginal effects of different interventions on participation, and therefore physical activity levels, using longitudinal data and sophisticated modelling techniques.

### Conclusion

In England in December 2018, 69% of the population lived within 5 km of a parkrun event. Creating 200 new events in the recommended (optimal) green spaces would further improve access, increasing this to 82%. Contrary to our expectation, we find that geographic access is slightly better for those living in more deprived communities. Given that participation rates are generally lower in deprived areas, improving access alone seems unlikely to significantly reduce inequalities in participation and physical activity. To design more effective strategies to improve engagement from deprived communities, a deeper understanding of the barriers to taking part in mass participation physical activity events is needed.

## Author statements

### Acknowledgements

The authors would like to thank the parkrun research board and parkrunUK for their support. The authors are also grateful to Ravi Maheswaran and Farouk Umar from the University of Sheffield, for helpful discussions of the methods in this article. The usual disclaimer applies.

### Ethical approval

Ethical approval was obtained from the Sheffield Hallam University Ethics Committee (ER10776545). The authors did not collect any personal information but only used aggregate secondary data. The parkrun Research Board approved this research project, and four of its members (A.M.B., H.Q., E.G. and S.J.H.) were actively involved in the interpretation of findings and writing of this manuscript.

### Funding

This work was supported by funding from 10.13039/100004440Wellcome [108903/B/15/Z and 108903] and the 10.13039/501100000858University of Sheffield. The funders had no role in study design; in the collection, analysis and interpretation of data; in the writing of the report; or in the decision to submit the article for publication.

### Competing interests

A.M.B., H.Q., E.G. and S.J.H. are members of the parkrun Research Board.
