# Supplementary material for: Multiple deprivation and geographic distance to community physical activity events — achieving equitable access to parkrun in England
Source: Public Health. 2020 Dec;189:48–53. doi: 10.1016/j.puhe.2020.09.002 (PMC7762722; doi:10.1016/j.puhe.2020.09.002)
Supplement: Multimedia component 1 [file mmc1.pdf]

Appendix

Table S1: List of all 200 recommended green spaces for setting up parkrun events to maximise geographic access.

Rank	Park name	District/City	Wtd. average distance	% Change in overall access	Park area (km2)	Longitude	Lattitude
1	Plumley Park	Weston-super-Mare	4.59	-0.534	0.12	-2.915	51.354
2	Gunby Park	Gunby, Spilsby	4.57	-0.490	0.47	0.195	53.179
3	unnamed	Birmingham	4.55	-0.446	0.13	-1.796	52.492
4	South Cliff Gardens	Scarborough	4.53	-0.435	0.14	-0.393	54.270
5	Market Gardens	Berwick-upon-Tweed	4.51	-0.411	0.16	-1.932	55.671
6	The Well Head Fields	Bourne	4.49	-0.365	0.10	-0.379	52.765
7	unnamed	Wellingborough	4.48	-0.332	0.17	-0.680	52.295
8	Thornham Park	Eye	4.46	-0.310	0.30	1.083	52.302
9	Ley Hill Recreation ...	Birmingham	4.45	-0.303	0.20	-1.979	52.421
10	unnamed	Swindon	4.44	-0.296	0.13	-1.782	51.587
11	unnamed	Wisbech	4.42	-0.284	0.14	0.155	52.668
12	unnamed	Leicester	4.41	-0.276	0.19	-1.341	52.558
13	Neatherd Moor	Dereham	4.40	-0.246	0.15	0.957	52.683
14	The Warren	Newquay	4.39	-0.246	0.13	-5.118	50.410
15	unnamed	Uttoxeter	4.38	-0.236	0.15	-1.863	52.959
16	Brookfields Park	Rotherham	4.37	-0.233	0.29	-1.317	53.510
17	Ellergreen Park	Kendal	4.36	-0.229	0.28	-2.771	54.349
18	The Park	Beverley	4.35	-0.228	0.13	-0.486	53.863
19	Poolfoot Farm Traini...	Thornton-Cleveleys	4.34	-0.228	0.14	-3.007	53.885
20	Kensington Gardens	Richmond	4.33	-0.228	1.07	-0.178	51.506
21	Burntwood Leisure Ce...	Burntwood	4.32	-0.228	0.10	-1.930	52.677
22	The Park	Market Drayton	4.31	-0.221	0.13	-2.463	52.913
23	Possingworth Park	Heathfield	4.30	-0.220	1.34	0.195	50.966
24	Osmaston Park	Derby	4.29	-0.220	0.13	-1.462	52.893
25	East Fen Common	Ely	4.28	-0.219	0.21	0.353	52.332
26	Brinton Park	Kidderminster	4.27	-0.216	0.11	-2.257	52.380
27	Kettlebrook Park	Tamworth	4.26	-0.212	0.35	-1.664	52.614
28	Parklands Leisure Ce...	Okehampton	4.25	-0.203	0.12	-3.995	50.734
29	Victoria Park	Tipton	4.25	-0.202	0.13	-2.061	52.525
30	Clarence Park	Bury	4.24	-0.201	0.23	-2.285	53.606
31	Link Common	Malvern	4.23	-0.200	0.20	-2.318	52.123
32	unnamed	Coalville	4.22	-0.194	0.13	-1.349	52.727
33	Chilton Fields Sport...	Stowmarket	4.21	-0.194	0.12	0.976	52.195
34	Queen's Park Sports ...	Chesterfield	4.20	-0.194	0.12	-1.433	53.233
35	Watermead Country Pa...	Leicester	4.20	-0.193	1.48	-1.104	52.686
36	Bromsgrove School Sp...	Bromsgrove	4.19	-0.187	0.11	-2.063	52.327
37	Stockley Country Par...	Uxbridge	4.18	-0.185	0.17	-0.455	51.516
38	Parnham Park	Beaminster	4.17	-0.185	0.14	-2.741	50.802
39	Mayon Green	Penzance	4.16	-0.186	0.14	-5.690	50.078
40	Bedlington Country P...	Bedlington	4.16	-0.183	0.64	-1.591	55.117
41	Monks-Neil Park	Stoke-on-Trent	4.15	-0.181	0.14	-2.187	53.069
42	Highdown Gardens	Worthing	4.14	-0.181	0.12	-0.441	50.824

43	Shobnall Leisure Com...	Burton-on-Trent	4.13	-0.179	0.29	-1.654	52.811
44	South Park	Oxford	4.13	-0.173	0.23	-1.229	51.751
45	unnamed	Bridgwater	4.12	-0.171	0.35	-2.955	51.157
46	Warley Park	Smethwick	4.11	-0.170	0.25	-1.981	52.473
47	Priory Park	Southend-on-Sea	4.11	-0.170	0.18	0.707	51.553
48	unnamed	Wantage	4.10	-0.170	0.11	-1.427	51.601
49	Knightshayes Park	Tiverton	4.09	-0.170	0.71	-3.479	50.921
50	Walton Hall Park	Liverpool	4.09	-0.168	0.54	-2.952	53.448
51	unnamed	Peterborough	4.08	-0.168	0.10	-0.276	52.595
52	Laithwaite Park	Wigan	4.07	-0.166	0.14	-2.658	53.541
53	Central Park	Plymouth	4.07	-0.163	0.68	-4.147	50.386
54	unnamed	Ilminster	4.06	-0.163	0.13	-2.916	50.923
55	Fox Hollies Park	Birmingham	4.05	-0.160	0.17	-1.821	52.437
56	Amesbury Park	Salisbury	4.05	-0.160	0.43	-1.789	51.174
57	One Leisure St Ives ...	Saint Ives	4.04	-0.156	0.16	-0.092	52.337
58	unnamed	Ilkley	4.03	-0.156	0.13	-1.729	53.882
59	Mandale Park	Rochdale	4.03	-0.157	0.19	-2.168	53.609
60	unnamed	Stoke-on-Trent	4.02	-0.157	0.33	-2.106	52.985
61	Fairway Sports Compl...	Sandown	4.01	-0.155	0.11	-1.166	50.657
62	Morden Hall Park	Morden	4.01	-0.155	0.40	-0.185	51.403
63	Langold Country Park	Worksop	4.00	-0.155	0.56	-1.127	53.371
64	Whittleford Park	Nuneaton	4.00	-0.155	0.50	-1.503	52.526
65	Chesham Moor	Chesham	3.99	-0.154	0.14	-0.599	51.693
66	Memorial Park	Willenhall	3.98	-0.154	0.14	-2.063	52.587
67	Philips Park	Manchester	3.98	-0.154	0.12	-2.194	53.488
68	Audley Park	Saffron Walden	3.97	-0.152	0.86	0.227	52.021
69	High Tor Recreation ...	Matlock	3.97	-0.150	0.19	-1.555	53.129
70	unnamed	Bristol	3.96	-0.150	0.15	-2.763	51.473
71	Wyken Croft Nature P...	Coventry	3.95	-0.150	0.25	-1.454	52.425
72	Scrivelsby Park	Woodhall Spa	3.95	-0.149	1.09	-0.097	53.176
73	unnamed	Thame	3.94	-0.146	0.12	-0.993	51.744
74	Victoria Park	Bristol	3.94	-0.145	0.21	-2.586	51.440
75	Grin Low And Buxton ...	Buxton	3.93	-0.145	0.42	-1.925	53.247
76	Decoy Country Park	Newton Abbot	3.92	-0.145	0.54	-3.603	50.518
77	unnamed	Driffield	3.92	-0.145	0.17	-0.452	53.997
78	Furzton Lake	Milton Keynes	3.91	-0.144	0.38	-0.763	52.014
79	Tynedale Park	Corbridge	3.91	-0.144	0.13	-2.011	54.966
80	unnamed	Wirral	3.90	-0.143	0.11	-3.015	53.326
81	Ampthill Park	Bedford	3.90	-0.143	0.63	-0.502	52.035
82	Woolten Woods And Ca...	Liverpool	3.89	-0.143	0.21	-2.868	53.369
83	Broome Heath	Bungay	3.89	-0.143	0.29	1.455	52.470
84	Manor Park	Sidmouth	3.88	-0.141	0.15	-3.235	50.726
85	Waldershare Park	Dover	3.87	-0.142	0.25	1.277	51.188
86	The Edge	Haslemere	3.87	-0.142	0.17	-0.746	51.095
87	Haden Hill Park	Cradley Heath	3.86	-0.139	0.37	-2.063	52.463
88	Cusworth Park	Doncaster	3.86	-0.139	0.22	-1.175	53.528
89	Marl Pits Sports Com...	Rossendale	3.85	-0.139	0.17	-2.269	53.705
90	Bowness Gardens	Bristol	3.85	-0.138	0.14	-2.606	51.497
91	Astley Park	Chorley	3.84	-0.138	0.45	-2.642	53.658

92	unnamed	Bournemouth	3.84	-0.136	0.14	-1.895	50.747
93	unnamed	Newcastle upon Tyne	3.83	-0.133	0.14	-1.678	54.986
94	Church Green	Attleborough	3.83	-0.132	0.15	1.041	52.480
95	Clee Fields	Grimsby	3.82	-0.132	0.17	-0.057	53.555
96	King Edward VII Park	Wembley	3.82	-0.130	0.10	-0.294	51.557
97	Birchall Playing Fie...	Leek	3.81	-0.129	0.12	-2.024	53.093
98	unnamed	Gravesend	3.81	-0.127	0.15	0.352	51.419
99	Knights Grange Sport...	Winsford	3.80	-0.127	0.77	-2.544	53.200
100	Saltburn Valley	Saltburn-by-the-Sea	3.80	-0.127	0.23	-0.970	54.579
101	Sunnyhill Park	Edgware	3.79	-0.126	0.22	-0.228	51.596
102	unnamed	Manchester	3.79	-0.126	0.34	-2.377	53.446
103	Mote Park	Maidstone	3.78	-0.126	1.75	0.547	51.263
104	New Park	Huddersfield	3.78	-0.126	0.18	-1.666	53.568
105	Upton Meadow	Wirral	3.77	-0.126	0.16	-3.104	53.380
106	unnamed	New Malden	3.77	-0.125	0.11	-0.269	51.393
107	Sullington Warren	Pulborough	3.76	-0.125	0.26	-0.439	50.919
108	Calthorpe Park	Fleet	3.76	-0.125	0.14	-0.850	51.277
109	Butterley Park	Ripley	3.75	-0.125	0.94	-1.380	53.054
110	Kings' Park	Retford	3.75	-0.125	0.10	-0.946	53.321
111	Montreal Park	Sevenoaks	3.75	-0.124	0.18	0.166	51.277
112	Parsloes Park	Dagenham	3.74	-0.124	0.60	0.135	51.544
113	Barton's Point	Sheerness	3.74	-0.123	0.31	0.790	51.436
114	Yeovil Recreation Ce...	Yeovil	3.73	-0.122	0.17	-2.638	50.953
115	Hall Park	Rushden	3.73	-0.123	0.14	-0.598	52.285
116	Leegomery Pools	Telford	3.72	-0.122	0.11	-2.496	52.708
117	The Park	Malton	3.72	-0.122	1.23	-0.925	54.121
118	unnamed	Boldon Colliery	3.71	-0.121	0.12	-1.450	54.951
119	Stanmore Country Par...	Bridgnorth	3.71	-0.121	0.16	-2.378	52.531
120	Lyme and Wood Pits C...	Saint Helens	3.70	-0.120	0.99	-2.653	53.462
121	Heacham Park	King's Lynn	3.70	-0.120	0.18	0.497	52.914
122	Paddington Recreatio...	Wembley	3.70	-0.120	0.11	-0.189	51.530
123	Beacon Park	Lichfield	3.69	-0.120	0.29	-1.837	52.684
124	unnamed	Birmingham	3.69	-0.120	0.12	-1.839	52.481
125	Queen's Park	Bolton	3.68	-0.120	0.20	-2.443	53.579
126	Seaton Valley Countr...	Torpoint	3.68	-0.120	0.58	-4.387	50.375
127	Chennell Park	Tenterden	3.67	-0.120	0.20	0.676	51.073
128	Southrepps Common	Lower Street, Norwich	3.67	-0.120	0.11	1.361	52.865
129	Test Park	Southampton	3.66	-0.119	0.14	-1.468	50.928
130	Jubilee Meadows	Crook	3.66	-0.119	0.16	-1.681	54.706
131	Tiptree Heath	Colchester	3.66	-0.119	0.21	0.731	51.799
132	Plumstead Common	Welling	3.65	-0.119	0.38	0.099	51.481
133	Marston Park	Frome	3.65	-0.119	0.93	-2.341	51.202
134	unnamed	Hassocks	3.64	-0.119	0.13	-0.164	50.946
135	Mill Green Nature Pa...	Cannock	3.64	-0.118	0.28	-2.015	52.689
136	Knighton Park	Leicester	3.63	-0.118	0.34	-1.106	52.599
137	Sluice Common	Downham Market	3.63	-0.115	0.12	0.367	52.583
138	The Lawn	Swindon	3.63	-0.114	0.36	-1.766	51.551

139	American Garden	Camberley	3.62	-0.114	0.25	-0.769	51.337
140	Streatlam Park	Barnard Castle	3.62	-0.112	0.35	-1.863	54.570
141	unnamed	Kingston upon Hull, Hull	3.61	-0.111	0.15	-0.368	53.776
142	The Park	Pocklington, York	3.61	-0.111	0.13	-0.756	53.930
143	Sports Centre and Me...	Stamford	3.61	-0.111	0.11	-0.472	52.656
144	unnamed	Pontefract	3.60	-0.110	0.15	-1.290	53.599
145	Howe Bridge Leisure ...	Manchester	3.60	-0.109	0.18	-2.502	53.514
146	unnamed	Bassingbourn, Royston	3.59	-0.109	0.14	-0.034	52.046
147	Jubilee Park	Doncaster	3.59	-0.109	0.11	-0.994	53.582
148	Wincombe Park	Shaftesbury	3.59	-0.109	0.18	-2.171	51.016
149	Daisy Farm Park	Birmingham	3.58	-0.108	0.11	-1.873	52.405
150	Oare Gunpowder Works...	Faversham	3.58	-0.107	0.16	0.875	51.325
151	unnamed	Crowland, Peterborough	3.57	-0.107	0.10	-0.161	52.674
152	unnamed	New Milton	3.57	-0.107	0.10	-1.669	50.747
153	Chafford Gorges Natu...	Grays	3.57	-0.107	0.26	0.302	51.488
154	Caythorpe Court	Grantham	3.56	-0.106	0.20	-0.571	53.022
155	unnamed	Stroud	3.56	-0.106	0.88	-2.218	51.733
156	American Gardens	Bodmin	3.56	-0.105	0.13	-4.763	50.503
157	Allesley Park Golf C...	Coventry	3.55	-0.105	0.12	-1.555	52.419
158	Lammas Lands	Godalming	3.55	-0.105	0.30	-0.601	51.189
159	unnamed	Liverpool	3.54	-0.104	0.11	-3.051	53.564
160	unnamed	Leatherhead	3.54	-0.104	0.13	-0.332	51.290
161	Brockhill Country Pa...	Hythe	3.54	-0.104	0.22	1.067	51.080
162	West Ham Park	Ilford	3.53	-0.103	0.26	0.020	51.539
163	Wassand Park	Hornsea	3.53	-0.103	0.42	-0.218	53.898
164	Humberstone Park	Leicester	3.53	-0.103	0.12	-1.083	52.639
165	Millbrook Park Mille...	Liverpool	3.52	-0.103	0.15	-2.891	53.487
166	Berrington Park	Leominster	3.52	-0.103	1.10	-2.719	52.265
167	Lily Hill Park	Bracknell	3.52	-0.103	0.24	-0.724	51.414
168	Naworth Parks	Brampton	3.51	-0.103	2.98	-2.684	54.957
169	unnamed	Immingham	3.51	-0.103	0.11	-0.200	53.615
170	Bennett's Park	Halstead	3.50	-0.103	0.24	0.631	51.964
171	Little Stoke Park	Bristol	3.50	-0.103	0.12	-2.560	51.528
172	Longdales Park	Nettleham, Lincoln	3.50	-0.103	0.13	-0.478	53.260
173	Southbury Leisure Ce...	Enfield	3.49	-0.103	0.38	-0.062	51.655
174	Hatch Grange	Southampton	3.49	-0.103	0.15	-1.336	50.930
175	Elmers Green Common	Skelmersdale	3.49	-0.102	0.22	-2.762	53.552
176	Kilburn Park	Thirsk	3.48	-0.102	0.22	-1.240	54.209
177	Murton Recreation Gr...	Seaham	3.48	-0.102	0.13	-1.389	54.812
178	Norbury Park	Thornton Heath	3.48	-0.102	0.11	-0.117	51.413
179	Fairbank Plantation	Carnforth	3.47	-0.102	0.11	-2.599	54.207
180	Sandbach United Foot...	Sandbach	3.47	-0.101	0.10	-2.382	53.138
181	Shobrooke Park	Crediton	3.47	-0.100	0.75	-3.626	50.799
182	Tadley Common	Tadley	3.46	-0.100	0.36	-1.127	51.356
183	Riverside Garden Par...	Horley	3.46	-0.100	0.11	-0.167	51.163
184	unnamed	Preston	3.45	-0.100	0.11	-2.734	53.796
185	unnamed	Calne	3.45	-0.099	0.10	-1.999	51.427

186	Mary Stevens Park	Stourbridge	3.45	-0.099	0.12	-2.149	52.448
187	Harvey Hadden Sports...	Nottingham	3.44	-0.098	0.17	-1.218	52.967
188	unnamed	Lancaster	3.44	-0.098	0.11	-2.820	54.064
189	Colemere	Ellesmere	3.44	-0.098	0.49	-2.842	52.893
190	Highmoor Park	Wigton	3.43	-0.098	0.34	-3.141	54.818
191	Lido Park Droitwich	Droitwich	3.43	-0.098	0.15	-2.143	52.263
192	unnamed	Brighton	3.43	-0.098	0.13	0.014	50.867
193	unnamed	Salford	3.42	-0.098	0.22	-2.296	53.497
194	Holywells Park	Ipswich	3.42	-0.098	0.18	1.174	52.047
195	Odd Down Sports Grou...	Bath	3.42	-0.097	0.17	-2.373	51.361
196	St Helen's Wood	Hastings	3.41	-0.097	0.40	0.580	50.878
197	Anstey Park	Alton	3.41	-0.097	0.19	-0.962	51.160
198	unnamed	Ruislip	3.41	-0.097	0.12	-0.407	51.561
199	Langdon Hills Countr...	Stanford-le-Hope	3.40	-0.097	1.71	0.436	51.545
200	Bexhill Down	Bexhill-on-Sea	3.40	-0.096	0.16	0.464	50.847
